# Homochirality in biomineral suprastructures induced by assembly of single-enantiomer amino acids from a nonracemic mixture

**DOI:** 10.1038/s41467-019-10383-x

**Published:** 2019-05-24

**Authors:** Wenge Jiang, Dimitra Athanasiadou, Shaodong Zhang, Raffaella Demichelis, Katarzyna B. Koziara, Paolo Raiteri, Valentin Nelea, Wenbo Mi, Jun-An Ma, Julian D. Gale, Marc D. McKee

**Affiliations:** 1 Department of Chemistry, Tianjin Key Laboratory of Molecular Optoelectronic Sciences, and Tianjin Collaborative Innovation Center of Chemical Science & Engineering, Tianjin University, Tianjin, P. R. China 300072; 2 Faculty of Dentistry, McGill University, Montreal, QC Canada H3A 0C7; 3 School of Chemistry and Chemical Engineering, Shanghai Jiao Tong University, Shanghai, P. R. China 200240; 4 Curtin Institute for Computation, The Institute for Geoscience Research (TIGeR), and School of Molecular and Life Science, Curtin University, GPO Box U1987, Perth, WA 6845 Australia; 5 Tianjin Key Laboratory of Low Dimensional Materials Physics and Preparation Technology, School of Science, Tianjin University, Tianjin, P. R. China 300354; 6 Department of Anatomy and Cell Biology, McGill University, Montreal, QC Canada H3A 0C7

**Keywords:** Biomineralization, Biomineralization, Organic-inorganic nanostructures

## Abstract

Since Pasteur first successfully separated right-handed and left-handed tartrate crystals in 1848, the understanding of how homochirality is achieved from enantiomeric mixtures has long been incomplete. Here, we report on a chirality dominance effect where organized, three-dimensional homochiral suprastructures of the biomineral calcium carbonate (vaterite) can be induced from a mixed nonracemic amino acid system. Right-handed (counterclockwise) homochiral vaterite helicoids are induced when the amino acid l-Asp is in the majority, whereas left-handed (clockwise) homochiral morphology is induced when d-Asp is in the majority. Unexpectedly, the Asp that incorporates into the homochiral vaterite helicoids maintains the same enantiomer ratio as that of the initial growth solution, thus showing chirality transfer without chirality amplification. Changes in the degree of chirality of the vaterite helicoids are postulated to result from the extent of majority enantiomer assembly on the mineral surface. These mechanistic insights potentially have major implications for high-level advanced materials synthesis.

## Abstract (web summary)

Induction of complex homochiral architectures by chiral transformation in a mixed enantiomer system has remained largely elusive. Here, the authors report a chirality dominance effect which induces homochiral suprastructures of calcium carbonate by a mixed, heterochiral nonracemic amino acid enantiomer system.

## Introduction

Nature displays many chiral hierarchical structures across different length scales, ranging from small molecules including amino acids and sugars^1^, to biomacromolecules such as collagen and tobacco mosaic virus^2,3^, and to complex biomineralized helical gastropod shells^4^. All these chiral moieties contain only one of two possible enantiomers, where enantiomeric pairs differ only in that their two structures are nonsuperimposable mirror images of each other^1^. Only pure enantiomeric (homochiral) structures for materials and medicinal drugs tend to be active in having their desired optical, mechanical, and therapeutic properties, whereas enantiomeric mixtures can have reduced activity, be inactive, or even biologically toxic (such as the drug thalidomide, causing birth defects)^5–7^. Homochiral selection events are also considered to be important to the origin of life^1^, and they can also have significant impact on the activity of different life forms. For example, homochiral spiraling snail shells provide benefit for copulation and thus species propagation^4^. Understanding how homochiral status can be achieved from initially mixed heterochiral enantiomeric conditions is therefore critical for many reasons^8,9^. Given that the transmission of chirality from the primary structure of molecular subunits to higher-level structures is responsible for creating different functionalities in complex hierarchical materials and biological organisms^10–13^, there is considerable interest in knowing how to preferentially favor the formation of one chirality over the other in mixed enantiomer systems^14^.

In the past several decades, for lower-level assembly structures of simple organic molecules, several models describe how to amplify a single chirality by chiral transition from a mixture of different primary enantiomers, such as the so-called “sergeants and soldiers” effect and the “majority rule” effect, as well as deracemization (or chiral self-sorting)^15–21^. Raval and co-workers have shown how configurational entropy effects in the disordered state affect self-organization, leading to the preferential assembly of the majority enantiomer to form homochiral conglomerate domains^13^. In contrast, for higher-level functional three-dimensional (3D) chiral suprastructures—such as found in biology and exemplified by biomineralized spiraling gastropod shells—inorganic/organic biocomposite materials have highly complex hierarchical structures. How complex 3D homochiral architecture is induced by chiral transformation using simple chiral organic molecules in a mixed enantiomer system has remained largely elusive. Although some progress has been made in showing that the handedness of organic molecules—including amino acids and DNA—can transfer their chiral property to materials such as biominerals and artificial inorganic catalysts^22–26^, there are no firm rules that predict whether the assembly of larger-scale homochiral suprastructure will be guided by the actions of the majority enantiomer, or whether heterochiral structure will result from nonracemic status in a mixed organic enantiomer system.

Enantiomers of the acidic amino-acid aspartic acid (Asp)—a particularly abundant acidic residue of biomineralization-regulating proteins—can transfer their chirality to achiral inorganic materials to form chiral architectures^24–27^; this transfer process has been proposed to play an important role in the origin of Nature’s homochiral biomolecules, where they may have assembled and organized on mineral surfaces in prebiotic conditions^28,29^. In addition, it has also been found that Asp oligomers [poly(aspartate)] and Asp-rich polymers can induce helical calcium carbonate vaterite structure^30,31^, and the chirality of helical vaterite structure is dependent on the chirality of Asp-rich polymer^31^. This chirality transfer in vaterite is similar to the chiral doping of nematic liquid crystals, where a simple chiral molecule/dopant can endow achiral nematic liquid crystals with macroscopic chiral helical structure, and provide new applications^32^. Vaterite is a polymorph of calcium carbonate that, in certain biological systems, can form chiral, hierarchically organized suprastructures as induced by chiral biomolecules for the hardened tissues of terrestrial and marine organisms^33–35^, and even in chiral pathologic otoconia of the human inner ear^36^.

Here, we report that uniform, helicoidal homochiral suprastructures of calcium carbonate (vaterite) are formed in the presence of nonracemic mixtures of acidic amino-acid Asp enantiomers. The degree of chirality (as defined by chirally oriented platelet density in biomimetic vaterite helicoids) is dependent on the critical assembly of majority enantiomer domains (of Asp) on the mineral surface as established from a mixed enantiomer system. Most strikingly, and in contrast to previous understanding^1,13,37^, this occurs without any chiral amplification of Asp within the homochiral vaterite helicoids, as the incorporated l-:d-Asp ratio is maintained at the same ratio as in the original aqueous solution from which the helicoids initially formed. From the initial events of this biomimetic mineral growth process in a nonracemic system, a pathway of chirality transmission is established that results in the formation of a complex homochiral biomimetic structure.

## Results

### Homochiral helicoids from a nonracemic enantiomer mixture

Under single-enantiomer conditions, l- and d-Asp each can endow what would otherwise be achiral calcium carbonate vaterite aggregate structure (where there is no additive) with chiral characteristics in which induction of oppositely chiral suprastructured vaterite helicoids (also referred to as toroids) are established by a nano-tilting growth mechanism^24^. In brief, vaterite crystallization in supersaturated solution of Ca^2+^ and CO_3_^2−^ with added chiral amino-acid involves adsorption of l- or d-Asp onto the vaterite surface of the “mother“ hexagonal nanoparticle to break what would otherwise be (without added amino acid) a perfectly oriented nucleation/attachment mechanism for the consequential “daughter” hexagonal nanoparticle leading to achiral vaterite growth. When Asp is added, the intervening adsorbed chiral Asp leads to a slight, misaligned counterclockwise or clockwise tilt angle of 4° between the nanoparticles in each platelet in the presence l- or d-Asp, respectively. With further replication and extension of this tilting effect induced by l- or d-Asp during vaterite platelet formation and growth, curved-edge, oriented counterclockwise or clockwise platelets arise from the helicoid surface. With further growth of the helicoidal structure, additional counterclockwise, or clockwise platelets arise to generate an initial, counterclockwise or clockwise helicoid in the presence of l- or d-Asp (Supplementary Figure 1). Extending from these initial observations, we examined the effects of a nonracemic solution system having both l- and d-Asp enantiomers in which one enantiomer of Asp is in excess of the other, referred to as enantiomeric excess of l-Asp (e.e._L_) as defined in equation 1:

1 $${\mathrm{e}}.{\mathrm{e}}._{\mathrm{L}} = \frac{{\left( {\theta _{\mathrm{L}} - \theta _{\mathrm{D}}} \right)}}{{(\theta _{\mathrm{L}} + \theta _{\mathrm{D}})}} \times 100{\mathrm{\% }}$$

where *θ*_L_ and *θ*_D_ are the concentrations of l- and d-enantiomer of Asp in the original solution mixture. In the current study, the total concentration of Asp enantiomers was kept constant at 20 mm, and the formulae e.e._L_ > 0, e.e._L_ = 0, and e.e._L_ < 0 represent the initial solution conditions of l-Asp being in the majority, of equal racemic balance (50:50), and of d-Asp being in the majority, respectively.

**Fig. 1:**
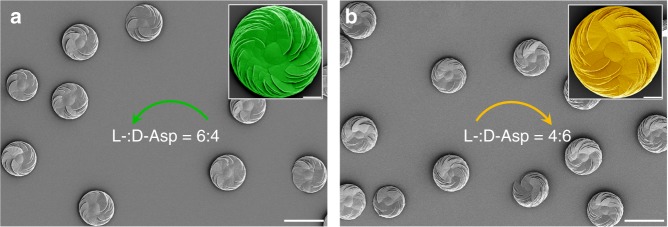
Homochiral vaterite helicoids induced in the presence of nonracemic mixtures of l- and d-enantiomers of amino acid. SEM images of chiral vaterite helicoids after 2 days growth in the presence of 20 mm total Asp enantiomer mixture consisting of 12 mm l-Asp and 8 mm d-Asp (6:4 ratio, e.e._L_ = 20%), showing identical counterclockwise orientation of vaterite platelets (green arrow, **a**). Conversely, 8 mm l-Asp and 12 mm d-Asp (4:6 ratio, e.e._L_ = −20%) produces identical clockwise orientation of the platelets (yellow arrow, **b**). Insets: pseudocolored single chiral vaterite helicoids at higher magnification for demonstrating platelet structure and orientation

In a racemic solution of equal parts l-Asp and d-Asp, only round symmetric achiral vaterite structures were observed without any readily identifiable accumulation of oriented platelets (Supplementary Figure 2). However, in the nonracemic condition where one Asp enantiomer is in excess of the other, slightly inclined platelets appeared at the vaterite surface and with growth evolution developed into a chiral, helicoid-shaped, and hierarchically organized suprastructure. Importantly, under this condition, all the precipitated vaterite helicoids were uniformly homochiral without exception (Fig. 1, and Supplementary Figure 1). When l-Asp was in slight excess (e.e._L_ = 20%), only counterclockwise platelets comprised the vaterite helicoids over 2 days of growth (green arrow, Fig. 1a, consistent with the effects of pure l-Asp); whereas when d-Asp was slightly in excess (e.e._L_ = −20%), only clockwise platelets comprised the vaterite helicoids (yellow arrow, Fig. 1b, consistent with the effects of pure d-Asp). From additional experiments, it is worth noting that these homochiral vaterite helicoids having all platelets uniformly oriented in a counterclockwise fashion were obtained in a nearly racemic solution with e.e._L_ as low as 5% (no oriented platelets formed on the surface of vaterite helicoid in 10 days if the e.e._L_ was < 5%, the results being similar to that observed under racemic conditions) (Supplementary Figure 3). To the best of our knowledge, this is first example showing that an initially nearly racemic mixture of enantiomers can give rise to uniform homochirality, as shown here for a 3D biomineral suprastructure.

### Maintenance of enantiomer ratio in helicoids and solution

**Fig. 2:**
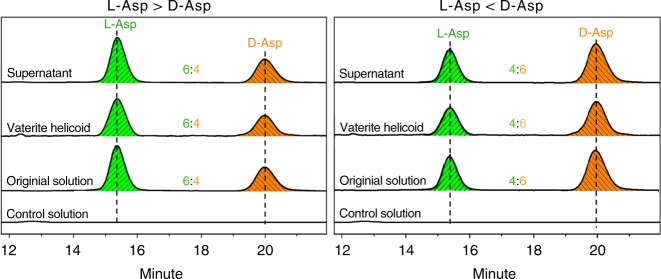
The ratio of l- to d-enantiomers incorporated into homochiral helicoids is maintained from the original imbalanced solution. RP-HPLC profiles collected from control calcium carbonate growth solution in the absence of Asp, and from the initial nonracemic Asp solution (l- and d-Asp having a ratio of 6:4 in the left panel, and conversely 4:6 in the right panel) prior to helicoid formation (two bottom spectra in each panel). HPLC profiles of l- and d-Asp enantiomers collected from dissolved homochiral vaterite helicoids, and from their respective supernatants after helicoid formation (two upper spectra in each panel)

Given that the amplification of molecular enantiomeric excess and even a homochiral adsorbate of one enantiomer can be achieved on a crystalline surface^13,37^, it seemed reasonable at the outset of this study to expect that in the case of enantiomeric Asp excess exposed to growing vaterite, that either only a single enantiomer of Asp would be incorporated into the homochiral vaterite helicoids, or that the ratio between incorporated l- and d-Asp should be increased compared with the initial imbalanced solution ratio. However, both these hypotheses proved invalid in our growth experiments with Asp and vaterite. Quantitative analysis, by reverse-phase, high-performance liquid chromatography (RP-HPLC) using a chiral column, of incorporated Asp enantiomers into the homochiral vaterite helicoids showed that both l- and d-Asp were incorporated, and that the ratio between them was maintained after vaterite growth, being equal to that of the original imbalanced starting solution, (Fig. 2, and Supplementary Table 1). For example, when the starting ratio of 6:4 l- to d-Asp (e.e._L_ 20%) was present in the original imbalanced solution, this same ratio was precisely maintained when the amino acids were extracted from the counterclockwise homochiral vaterite helicoids following their dissolution with acid (a brief sodium hypochlorite wash was first used to remove weakly bound surface Asp enantiomers; see details in Methods section). Furthermore, the same 6:4 ratio of l- to d-Asp remained in the final supernatants after vaterite helicoid formation and growth. Taken together, both the SEM images and the results of the RP-HPLC indicate that despite there being no chiral amplification of amino acid enantiomer in the helicoids (Figs. 1 and 2, and Supplementary Table 1), homochiral organization of biomineral suprastructure can be established (dominated) by the chirality of the majority enantiomer.

### Regulation of platelet density within chiral helicoids

In the pure l-Asp system, the density of counterclockwise platelets on the helicoid surface increased nonlinearly with increasing concentration of enantiomer (Supplementary Figure 4). In addition to this, the density of the platelets (*D*, i.e. the platelet number per square of micrometer area of the helicoid surface) was proportional to the square of the l-Asp concentration as shown:

2 $$D = a_{pure\;{\mathrm{L}} - {\mathrm{Asp}}}\left( {C_{{\mathrm{L}} - {\mathrm{Asp}}}} \right)^2$$

where the slope *a*_pure_ is 0.2 µm mm^−2^.

**Fig. 3:**
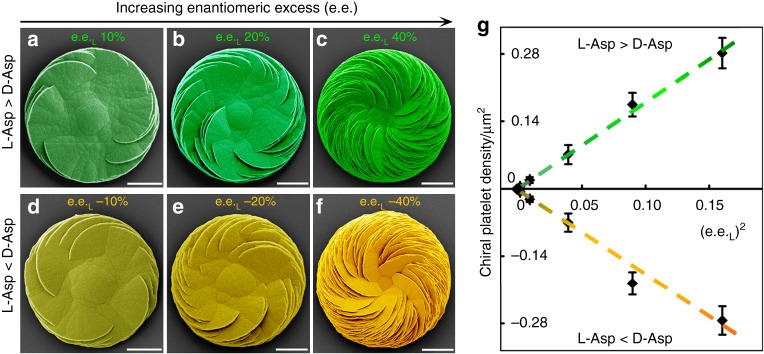
Relationship between the density of platelets within a chiral vaterite helicoid to enantiomeric excess. **a**–**f** Representative SEM images of vaterite helicoids showing chiral vaterite platelets having only counterclockwise or clockwise orientation when either l- or d-Asp is the majority enantiomer under nonracemic solution conditions, respectively. The number of counterclockwise and clockwise platelets increases with increasing enantiomeric excess of l- or d-Asp (green and yellow, pseudocolored, respectively). **g** Plot showing a linear proportionality relationship between the density of homochiral platelets and the square of enantiomeric excess (e.e._L_)^2^, which indicates a nonlinear increase in oriented platelets with increasing enantiomeric excess (e.e.) as shown in Supplementary Figure 5 (“+” and green colors are arbitrarily assigned to counterclockwise-oriented platelets induced by l-Asp, and “−” and yellow colors to clockwise-oriented platelets induced by d-Asp). Error bar represents standard error of mean (Scale bar: 10 μm)

Under imbalanced enantiomer conditions of Asp, although the chiral orientation of the platelets and the helicoid as a whole were fixed (from domination by the majority enantiomer of Asp), it was observed that the chiral platelet density on the helicoids quickly increased nonlinearly with increasing enantiomeric excess in a manner similar to that observed when pure l-Asp concentration alone increased (Supplementary Figure 5). The chirally oriented platelet density (*D*) of the helicoids was proportional to the square of enantiomeric excess (e.e._m_) or the excessive concentration of majority enantiomer after neutralization with minority enantiomer (*θ*_e.c_), rather than to its absolute concentration in the mixed original solution (Fig. 3g), which are given by:

3 $$D = a_{{\mathrm{e}}.{\mathrm{e}}.}(e.e._{\mathrm{m}})^2$$

4 $$D = a_{{\mathrm{e}}.{\mathrm{c}}.}(\theta _{{\mathrm{e}}.{\mathrm{c}}})^2$$

where the slopes *a*_*e.e*._ and *a*_*e.c*._ are 1.84 µm^−2^ and 0.0046 µm^−2^ mm^−2^, respectively.

Although the reason for this proportionality is not yet clear, it nonetheless demonstrates that the density of chiral platelets grows in a predictable manner, and this provides a powerful tool to control the platelet growth process by simply varying the concentration of the enantiomer in either homochiral solution or in nonracemic solution of amino acids. Additionally, it is worth noting that the slope of 0.2 µm mm^−2^ in the pure enantiomer system is more than 40 times that of 0.0046 µm mm^−2^ in the mixed enantiomer system, which directly reveals that the increasing rate of chiral platelet density in a helicoid in the pure enantiomer system is much higher than that in the mixed enantiomer system. This great difference in the slope also suggests that the minority enantiomer can effectively inhibit chiral platelet formation induced by majority enantiomer in the mixed system.

### Assembly of Asp enantiomers incorporated into the helicoid

In biomineralization, self-assembly of incorporated biomolecules is a general strategy to regulate the crystallization and organization of inorganic mineral^2,38–43^, such as occurs in bones and teeth and other biomineralized structures. Furthermore, self-assembly of chiral molecules at interfaces is critical for transmitting chirality information from single chiral molecules to higher-level, complex chiral structures having specific functions^17,22,25,42–44^. Here, to examine the assembly of incorporated amino-acid enantiomers into homochiral vaterite helicoids, we applied micro-Raman spectroscopy. Although scanning tunneling microscopy can be used to characterize the assembly of chiral molecules on simple, flat conductive metal crystal surfaces^13,15^, Raman and infrared spectroscopies can provide ultra-high-resolution observations on the organization of molecules in various complex systems, including in biomaterials^25,45–48^. This is possible by virtue of the ability of these spectroscopies to obtain data independent of background substrate roughness and conductivity. Because of their extreme sensitivity in detecting changes in the stretching behavior of functional groups of molecules, changes in molecular assembly can be accurately probed by observing spectroscopic shifts of vibrational bands in functional groups^45–48^.

**Fig. 4:**
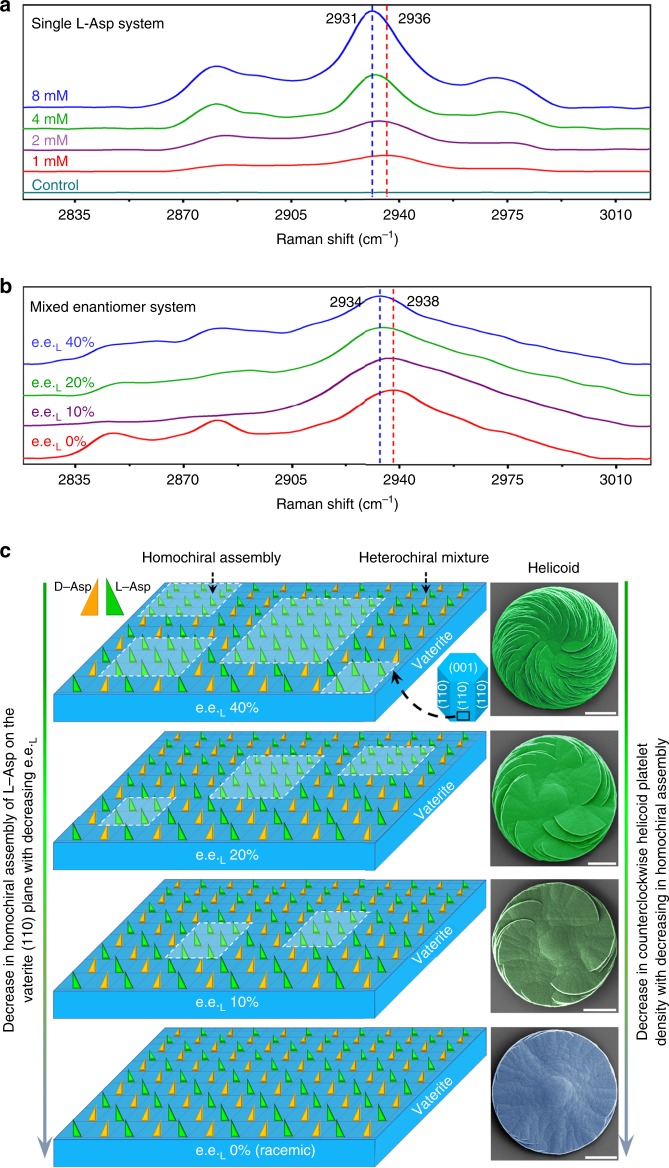
Mechanism of the formation of homochiral helicoids by the assembly of incorporated amino-acid enantiomers. **a** Micro-Raman spectra of C-H stretching bands of vaterite helicoids formed in the presence of only l-Asp at different concentrations in supersaturated calcium carbonate solution; **b** micro-Raman spectra of vaterite helicoids formed in the presence of a mixture of l-and d-Asp enantiomers (total Asp concentration equals 20 mm) at different e.e._L_. All micro-Raman spectra are normalized to the ν_1_ symmetric stretching peak of carbonate groups in vaterite helicoids at 1089 cm^−1^. **c** Schematic summary of the co-existence of heterochiral and homochiral domains on a vaterite crystal plane with spacing *d* = 3.6 Å, as identified by high-resolution transmission electron microscopy as shown in ref. ^25^. In a nonracemic system, a decrease in enantiomeric excess of excessive l-Asp (e.e._L_) leads to a decrease in homochiral assembly, which in turn results in a decrease in counterclockwise platelet density in the vaterite helicoids (SEM imaging). In a racemic system, equal amounts of l- and d-Asp are assembled across the full surface, and no effective homochiral assembly domains are formed, and thus no chiral morphology emerges (Scale bar: 10 μm)

In our vaterite helicoid growth system, the assembly behavior of the chiral enantiomers of Asp in the helicoids was identified by micro-Raman spectroscopy for both the single (pure, not mixed) enantiomer system and the enantiomeric mixed system (Fig. 4). In the pure l-Asp system, the normalized main C-H stretching bands of l-Asp at 2930–2940 cm^−1^ in micro-Raman spectra of counterclockwise vaterite helicoids increased in their intensity with increasing concentration of l-Asp in solution, demonstrating the concentration-dependent increase in l-Asp incorporation into the vaterite helicoids. More importantly, besides the intensity strength signal described above, there was a continuous concentration-dependent shift to lower frequency for the main C-H stretching bands of incorporated l-Asp, occurring from ~ 2936 cm^−1^ at 1 mm (red dashed line, Fig. 4a) to 2931 cm^−1^ at 8 mm (blue dashed line, Fig. 4a). This continuous shift shows that the level of assembly increased with increasing incorporated l-Asp enantiomer^45–48^, which leads to the increase in the density of counterclockwise-oriented platelets in homochiral vaterite helicoids (Supplementary Fig. 4).

Under mixed enantiomer conditions (l-Asp together with d-Asp), the presence of minority enantiomer d-Asp affects the assembly of the majority l-Asp enantiomer in the counterclockwise vaterite helicoids, as demonstrated by the shift back (like for the pure l-Asp alone condition) of the main C-H band of the incorporated l-Asp (Fig. 4b). More specifically in this regard, the main C-H stretch band of incorporated l-Asp (8 mm) alone at 2931 cm^−1^ (blue dashed line, Fig. 4a) was shifted forward to higher frequency at 2934 cm^−1^ in the case of majority l-Asp (with e.e._L_ 40%) in the mixed enantiomer system (blue dashed line Fig. 4b compared to blue dashed line Fig. 4a), despite both conditions having roughly the same net high Asp concentration.

The degradation of the assembly of majority l-Asp caused by minority d-Asp determines the density decrease of the corresponding counterclockwise platelets, which thus explains why the increasing rate of counterclockwise platelet density of the helicoids in the pure enantiomer system is higher than that in the mixed enantiomer system, as shown by the difference between the slopes of equations 2 and 4 (Fig. 3, and Supplementary Figure 4). In addition, the main C-H stretching peaks of helicoid-incorporated l-Asp enantiomer continuously shifted back to high frequency with an increase in minority d-Asp concentration (i.e., decrease of e.e._L_) (Fig. 4b), suggesting that the assembly of incorporated majority enantiomer l-Asp was decreased with the decreased e.e._L_ (i.e., an increase of minority d-Asp). Under the racemic (50:50) condition, the extent of assembly of homochiral domains of Asp enantiomers was the weakest, confirmed by the highest frequency peak being at 2938 cm^−1^ (red dashed line, Fig. 4b) rather than at 2931 cm^−1^, despite the fact that the net concentration of l-Asp was similarly high in both cases. Moreover, the total incorporation amount of l- and d-Asp into the helicoids was the same for all mixed enantiomer conditions, as demonstrated by the maintenance of the intensity of the main C-H stretch peak of Asp (Fig. 4b) (which corresponded to ~1.7% weight percent by RP-HPLC and calcium quantification). The two lower frequency bands of Asp at 2880 and 2846 cm^−1^ in panel 4b are from the vibrational symmetric stretch of the CH_2_ group in Asp^49,50^, that are prominent at the racemic e.e. 0% condition; these arise from the strong signals characteristically obtained by micro-Raman spectroscopy from flat surfaces (here the flat achiral vaterite disc, in panel 4c bottom-right micrograph) versus the rougher surfaces of the platelet-containing helicoids (Fig. 4c, right top three chiral vaterite helicoid micrographs). Conversely, where d-Asp replaced l-Asp as the majority enantiomer, the main C-H stretch peak of incorporated d-Asp enantiomer likewise continuously shifted to lower frequency, suggesting again that the assembly of majority enantiomer (here being d-Asp) in clockwise helicoids was enhanced with e.e._D_ increase. It is precisely this variation in the extent of assembly of the majority enantiomer that is responsible for the change in chiral platelet density as formed by the nano-tilting mechanism; this allows for thermodynamic stabilization in the mixed enantiomer solutions as shown in Fig. 3 (see details in Supplementary Discussions). Consistent with this notion our metadynamics simulation, circular dichroism (CD) and nuclear magnetic resonance (NMR) data all confirm that no homochiral pre-assembly of the amino acid alone occurs initially in aqueous solution (Supplementary Figures 6–9, Supplementary Table 2). Thus, we conclude that the homochiral assembly of Asp enantiomer occurs only on the solid vaterite crystalline surface where the mineral surface itself (lattice atoms) induces the assembly of homochiral domains^29,44^.

Ab initio calculations in vacuum show a strong preference for the formation of homochiral clusters (−26.5 kJ/mol for the fully deprotonated Asp species dominant at the experimental pH). As mentioned in the previous paragraph, this preference is not observed in water and this could be partly attributable to the clusters being highly hydrated and weakly bound (see details in Supplementary Discussions). When binding to the mineral surface, however, part of this water would be removed from the cluster, and this could be one of the reasons for the formation of homochiral clusters becoming more favorable at the vaterite surface. Indeed, molecular dynamics simulations show binding of Asp to vaterite via at least a single well-defined minimum (Supplementary Figure 9, and see Supplementary Discussions).

For the mixed, noninterconvertible enantiomers, besides the templating effect of the inorganic crystal surface to induce the homochiral assembly of pure enantiomers as described above, the behavior of molecular enantiomers can also be regulated by affecting maximal configurational entropy to disorganize enantiomers and form a heterochiral mixture with equal portions of the two enantiomers, for the maintenance of system stability^13^. This leads to the maintenance of the Asp enantiomer ratio in both homochiral vaterite helicoids and the original aqueous solution (Fig. 2). In the nonracemic system having excess l-Asp enantiomer, disorganized heterochiral mixture and homochiral assembly can co-exist on the vaterite crystal surface (Fig. 4c). With an increase in minority enantiomer d-Asp (i.e., a decrease in e.e._L_), more and more majority enantiomer l-Asp is neutralized by d-Asp, which therefore enhances the heterochiral mixture guided by maximal configurational entropy. In turn, the enhancement of a heterochiral mixture can lead to a decrease in homochiral assembly of pure l-Asp on the vaterite crystal surface as shown by micro-Raman spectroscopy (Fig. 4b), and thus a decrease in corresponding counterclockwise platelet density in homochiral vaterite helicoids (Fig. 3). When l-Asp is equal to d-Asp in the balanced racemic condition, the disorganized, heterochiral mixing of Asp enantiomers dominates and no effective homochiral assembly domain is formed on the vaterite crystal surface, which explains why no chiral property emerges (Figs. 3 and 4).

To provide additional evidence for the formation of chiral domains on calcium carbonate, we scanned the surface of freshly cleaved calcite by AFM after exposure to amino acid enantiomers—AFM can be used independently of the conduction properties of a substrate^51,52^. To investigate the aggregation of Asp enantiomers into domains on cleaved calcite, we added pure l-Asp alone, a nonracemic enantiomeric mixture (with e.e._L_ being 20%), and a racemic enantiomeric mixed system with equal parts of l-Asp and d-Asp (Supplementary Figure 10). In the case of exposure to only l-Asp, AFM visualization of the calcite surface showed extensive and large aggregated domains of homochiral l-Asp (Supplementary Figure 10b). However, with the addition of minority d-Asp into the system, the formation of homochiral domains of l-Asp decreased (Supplementary Figure 10c), and essentially no large aggregation domains were observed in the racemic system having equal parts l-Asp and d-Asp (Supplementary Figure 10d). The inhibitory effect of the minority d-Asp on the aggregation domains of the majority l-Asp observed on cleaved calcite by AFM was similar to that observed by micro-Raman spectroscopy in the vaterite helicoid system. Thus, this is additional evidence for the proposed model where the assembly of homochiral domains of majority l-Asp (whose extent is regulated by the amount of minority d-Asp) provides a plausible and consistent explanation for what we have noted in the formation of chiral vaterite helicoids. Although the homochiral assembly of single-enantiomer amino acids on the vaterite surface in a nonracemic system is our preferred mechanism for the creation of the homochiral hierarchical suprastructure seen in the vaterite helicoids, the possibility of other mechanisms cannot be entirely excluded, such as where a chiral single crystal can be induced by the selective regulation of certain crystal steps by chiral enantiomers of amino acids^26^.

## Discussion

This work demonstrates that when an imbalanced adsorption of two Asp enantiomers occurs on a vaterite mineral surface after growth in an imbalanced (nonracemic) enantiomer mixture, the chirality of the adsorbed majority amino acid enantiomer—acting through assembled homochiral domains—determines in a controllable and predictable manner the overall chirality of the organized supramolecular vaterite structure. Compared with previous studies mainly focusing on how to amplify a single chirality in lower-level (usually 1D or 2D) assembly structures of simple organic molecules from a mixed system, this work shows that higher-level complex 3D homochiral architectures can also be induced by chiral transformation using simple chiral organic molecules in a mixed enantiomer system. More importantly, differing from the dominant understanding of chiral amplification of organic molecules having enantiomer ratio change for homochiral structure synthesis, this study now shows that the assembly of the majority enantiomer in a mixed system can also induce homochirality in 3D suprastructures while maintaining the enantiomer ratio (i.e., no chiral amplification). Furthermore, our observations on the assembly of identical amino-acid enantiomers on a calcium carbonate mineral surface provides insight into one possible mechanism for mineral-mediated, chiral molecular concentration and organization, leading to biomolecular homochirality from a prebiotic, mixed enantiomer environment^29^. In addition, these findings also advance our understanding of mechanisms in biomineralization by which chiral biomineral growth (like in gastropod snails for example) might be guided by biomolecules, and they describe fundamental principles guiding the creation of organized and complex, higher-level functional chiral suprastructures, similar to those seen in various biological systems that mineralize.

## Methods

### Preparation of homochiral vaterite helicoids

The method for obtaining homochiral calcium carbonate vaterite helicoids in the presence of an nonracemic mixture of Asp enantiomers was similar to that used in our previous work^25^, with minor changes. In a 250 mL covered beaker, calcium carbonate was obtained by adding 50 mL of 3 mm Na_2_CO_3_ (Fisher Scientific, Canada) to 50 mL of a 3 mm CaCl_2_ (Fisher Scientific, Canada) solution containing total 40 mm mixture of l- and d-Asp, with the ratio between the two being varied (Sigma-Aldrich, Canada). Solution pH was adjusted to 10.5 ± 0.2 by the stepwise addition of 1 m NaOH at 20 °C. The final concentrations of CaCl_2_, Na_2_CO_3_ and the nonracemic mixture of l- and d-Asp were 1.5 mm, 1.5 mm, and 20 mm with variable enantiomeric excess ratios, respectively. The solution was maintained at room temperature without stirring during the entire growth period. When particulate material began to appear at the bottom of the beaker as observed by optical microscopy between 12 and 24 h, round glass coverslips (to facilitate removal and subsequent mineral analysis) were gently dropped to the bottom of the beaker. Periodically, after different reaction times, glass coverslips with attached chiral vaterite helicoids were removed from the beaker and washed gently and briefly three times with distilled water and then ethanol, and allowed to dry in a vacuum desiccator at room temperature before analysis.

### Quantification of calcium in chiral vaterite helicoids

The calcium concentration in acid-dissolved vaterite helicoids as grown directly on glass coverslips was quantified using a calcium assay kit (Sekisui Diagnostics Inc, PEI, Canada). In brief, homochiral vaterite helicoids grown in the presence of a mixture of l- and d-Asp at different ratios were dissolved in 100 μL of 10% acetic acid and the calcium content was spectrophotometrically quantified using the reaction kit reagents and a microplate reader measuring at 595 nm wavelength absorbance. The concentrations of calcium were established from standard curves using the biochemical assay kits and microplate reader spectrophotometric measurements.

### Reverse-phase high-performance liquid chromatography (RP-HPLC)

For the quantification of incorporated enantiomers of Asp into homochiral vaterite helicoids by RP-HPLC, all homochiral vaterite helicoid samples collected from glass coverslips on which they were grown were bleached in 12% w/v NaClO solution for 2 days before dissolution. This method effectively removes any surface-bound amino acids, as demonstrated during the characterization of biomolecules occluded within a range of biominerals^53^. The vaterite samples were rinsed three times with distilled water. The l- and d-enantiomers of Asp were then extracted from the helicoids by dissolving them in cold 1 m HCl for 10 min with a minimum of 20 μL HCl per mg of the helicoids. The dissolved solution samples were analyzed for l- and d-enantiomer of Asp using a 1100 Series LC System (Agilent, USA) and a chiral HPLC column of Chirex® 3126 (D)-penicillamine (4.6 × 250 mm, Phenomenex, USA), which directly separates amino-acid enantiomers by the chiral ligand selector, with ultraviolet detection being set at 254 nm. For this detection, the mobile phase was composed of 2 mm copper (II) sulfate in a mixed 2-propanol and water solution (95:5, v/v) at a flow rate of 1.0 mL/min at room temperature. The retention times of l- and d-Asp were ~15.3 min and 20.0 min, respectively. The concentrations of l- and d-Asp in various solutions were determined from respective standard curves, and reported by using the RP-HPLC peak areas of l- and d-Asp obtained from the chiral column^28^.

The composition of chiral acidic amino acids incorporated into the chiral vaterite helicoids was determined from calcium concentration measurements, and chiral acidic amino-acid concentration by RP-HPLC, after dissolution of the helicoids.

### Scanning electronic microscopy (SEM)

With calcium carbonate not being conductive, specimens were coated to reduce charging in the microscope by applying a 4-nm, electrically conductive layer of Pt using a sputter coater (Leica Microsystems EM ACE600, Vienna, Austria). Imaging was performed using a FEI Quanta 450 FE-ESEM (FEI Company, Hillsboro, OR, USA) operating at an acclerating voltage of 5 kV, and equipped with an Everhart-Thornley secondary electron detector.

### Micro-Raman spectroscopy

To measure chiral amino-acid incorporation from chiral vaterite helicoids, and to confirm the assembly behavior of incorporated amino-acid enantiomer into the helicoids formed both in both pure enantiomer system and in nonracemic mixture system, micro-Raman spectroscopy was performed on whole and intact helicoids using a Renishaw Via Raman microscope (Renishaw, Gloucestershire, UK) equipped with a holographic spectrometer and a Leica DM2500 M optical microscope (Leica Microsystems GmbH, Wetzlar, Germany). For this, all vaterite samples on glass coverslips were bleached with 12% w/v NaClO for 2 h to remove any weakly surface-bound amino acids, and then they were rinsed three times with distilled water and then ethanol, and allowed to dry in a vacuum desiccator for at least 7 days at room temperature before investigation to remove the water completely. The Raman spectra were recorded using a 514.5 nm argon laser as the excitation source with a laser spot size of *ca*. 2 µm, and an excitation power of 25 mW. The laser was focused through a × 50 objective having a numerical aperture of 0.75 on a single helicoid as grown previously on a glass coverslip. Each Raman spectrum was acquired typically for 20 s, and with five scans to minimize noise effects. Spectral reproducibility was confirmed by taking several spot analyses. All Raman spectra were obtained at room temperature using a spectral resolution of 1 cm^−1^, and a measurement range from 125 to 3205 cm^−1^. Calibration was performed by measuring the Raman spectrum of a silicon wafer. Data acquisition was performed by the software Renishaw WiRE 3.4 (Windows-based Raman Environment). All data were analyzed by the OriginLab 6.1 software.

### Circular dichroism (CD)

CD spectrometry measurements were performed with a J-815 spectropolarimeter (Jasco Inc., Easton, MD, USA). Measurements were run in the spectral range of 400–180 nm with samples (amino acids, in distilled water) placed in a 1-mm-thick, 150 µL capacity quartz cuvette. Measured parameters were as follows: sensitivity, 100 mdeg; data pitch 0.1 nm; scan mode, continuous; response, 1 s; scanning speed, 100 nm/min; accumulation, 5 with. Readout responses were millidegree (mdeg) units. The absolute values (in mdeg) of maxima and minima of positive and negative absorption bands were retained and used for plotting in Supplementary Figure 6.

### Nuclear magnetic resonance (NMR)

In order to verify the assembly of Asp enantiomers alone in aqueous solution, proton NMR (^1^H NMR) spectra of Asp enantiomers were performed in D_2_O, and recorded on a Bruker AV-400 (400 MHz) spectrometer. All chemical shifts are recorded in ppm on the δ scale, and the residual water in D_2_O was used as the internal reference (ref. δ_H_(H_2_O) = 4.79 ppm).

### Atomic force microscopy (AFM)

AFM images were collected at room temperature (20 °C) using a Multimode 8 AFM with a Nanoscope V controller (Bruker, USA). The (104) plane of cleaved calcite was examined in the tapping mode using an E-scanner in air. Scans were performed at rates of 0.1–2 Hz with a silicon V-shaped AFM probe on a cantilever having a spring constant *k* = 42 Nm^−1^ (TESPA-V2, Bruker). To reduce imaging artefacts, the tip force exerted on the surface was optimized by setting the amplitude set-point as high as possible.

Pure crystalline calcite (Iceland spar crystals), which were obtained from Chihuahua, Mexico (Sierra Madre Traders, Tucson, AZ), The calcite was cleaved using a razor blade, exposing the (104) plane. In total, 4 μl of 1 mm Asp aqueous solution with a known ratio of l- to d-Asp was carefully placed onto the large, cleaved calcite surface (~ 9 × 9 mm) under an optical microscope. The incubation period ranged from several seconds to a maximum of 2 min, then the solution was gently pipetted off and the sample was dried immediately by gentle exposure to a pure nitrogen gas stream.

### Computer simulation

Classical and ab initio molecular dynamics were used in this work; simulations were run using the LAMMPS and the CP2K codes^54,55^. The Plumed 2.4 plug-in was used for multiple-walker well-tempered metadynamics simulations^56–59^. Gas phase ab initio calculations on aspartate clusters were performed using the ORCA software package^59^. The full methodology and choice of parameters are reported in the Supplementary Methods.

The SPC/Fw water model^60^ was used and the parameters for calcium carbonate were taken from ref. ^61^. The force field for the aspartate anions was derived from GROMOS with intermolecular parameters obtained from the Automated Topology Builder and Repository^62,63^. The derivation of the parameters that describe the interactions of aspartate with water and calcium carbonate is described in the Supplementary Methods.

In order to probe the stability of the vaterite surface, geometry optimizations were performed in the presence of a continuum solvent model. This uses the COSMIC method as implemented in GULP, which is a variant of the COSMO methodology^64–66^. Additional details on this method and the parameters used in these calculations are reported in the Supplementary method.

## Supplementary information

Supplementary Information

## Data Availability

The data that support the findings of this study are available from the corresponding author (M.D.M.) upon reasonable request.
