# Supplementary material for: Homochirality in biomineral suprastructures induced by assembly of single-enantiomer amino acids from a nonracemic mixture
Source: Nat Commun. 2019 May 24;10:2318. doi: 10.1038/s41467-019-10383-x (PMC6534569; doi:10.1038/s41467-019-10383-x)
Supplement: Supplementary file 1 — Supplementary Information [file 41467_2019_10383_MOESM1_ESM.pdf]

Homochirality in biomineral suprastructures induced by assembly of single-enantiomer amino acids from a nonracemic mixture. Jiang et al.

Supplementary Methods

Computer simulation. Classical and *ab initio* molecular dynamics were used in this work. All classical simulations were run using the LAMMPS code with a time step of 1 fs and a Nosé-Hoover thermostat and barostat within the isothermal-isobaric ensemble with cubic boxes containing ~4,170 water molecules (corresponding to a cell length of ~49.9 Å)[1]. The relaxation times for thermostat and barostat were set to 0.1 and 1 ps, respectively. The surface calculations were run under similar conditions, for up to 70 ns, with a monoclinic cell size of 46.161 x 47.510 x ~81.5 Å and an angle of 108.92 degrees, corresponding to ~3,270 water molecules and 10 $CaCO_3$ layers. Multiple-walker well-tempered metadynamics, using the Plumed 2.4 plug-in[2-4], was used to explore the free energy profile of cluster aggregation in solution. The distance between the center of mass of aspartate and calcium was used as a collective variable. Simulations using the distance of the two carboxylate groups with calcium and the C-C-C-C torsional angle of aspartate as collective variables provide similar numerical results for the ion pair association. Having to explore only one collective variable significantly reduces the computational demands, at the cost of losing information regarding the atomic contribution of each separate functional group and of the cluster geometry. Gaussian functions with a width of 0.2 Å were deposited every 1 ps, with an initial height of k_BT. We used 50 parallel walkers (total simulation time of over 1 μs) and a bias factor of 5 to progressively reduce the heights of the Gaussians until convergence was achieved. All binding free energies quoted include correction to the standard state.

In order to probe the hydration structure of the aspartate anion in water, *ab initio*, molecular dynamics was performed using the program CP2K with the BLYP-D3 functional

based on the Gaussian planewave approach[5,6]. The core electrons and nuclei were described using GTH pseudopotentials with valence basis sets of TZ2P quality[7]. The electron density was described with an auxiliary basis set of planewaves with a cut-off of 400 Ry, while the self-consistent field calculation was performed using the orbital transformation algorithm[8]. Aspartate, with a net charge of -2, was placed in a cubic cell with a dimension of 13.088 Å containing 70 water molecules using an initial configuration derived from equilibrating with force field-based molecular dynamics. Simulations were run for up to 32 ps in the NVT ensemble using a time step of 0.5 fs and the CSVR thermostat. Here, an elevated temperature of 330 K was used to offset the known systematic over-structuring of water for the BLYP density functional, as with all GGAs, despite the inclusion of empirical Grimme-style dispersion corrections[9,10]. A similar procedure was adopted for the aspartate negative zwitterion, with a net charge of -1, except that the cubic box had a length of 13.774 Å, containing 85 water molecules, and the simulation length was 50 ps. Gas phase *ab initio* calculations on aspartate clusters were performed using the ORCA software package[11] at the ωB97X-D3/def2-TZVP level of theory with corrections for vibrational contributions at standard conditions.

The force field for the aspartate anions (L- and D-Asp^{2-}; L- and D-Asp^{1-}) was derived from GROMOS with intermolecular parameters obtained from the Automated Topology Builder (ATB) and Repository[12,13]. Due to the scarcity of experimental information regarding the water structuring around aspartic acid species, the interactions with water were then refitted to match the pair distribution function obtained from *ab initio* molecular dynamics; the parameters are reported in Supplementary Tables 3-5 and in Supplementary Figure 5 (below).

The SPC/Fw water model[14] was used and the parameters for calcium carbonate were taken from Ref 15. The interactions of aspartate with carbonate were derived from the methylammonium-carbonate interactions with the GROMOS force field as obtained from the ATB and Repository without change and from intermolecular carboxylate-carboxylate interactions of aqueous aspartate. The interactions between calcium and aspartate were derived from fitting of Buckingham potentials to gas phase quantum mechanical data for a Ca^{2+} ion interacting with acetate (to derive the Ca-carboxylate repulsion) and methyl amine (for the Ca-N repulsion). In addition, it was found to be necessary to add a short-range repulsive Lennard-Jones potential for the interaction between calcium and the carbon of the carboxylate group to prevent unphysical configurations from occurring in instances where the ion was above the plane of this functional group, as opposed to in the natural bidentate binding structure.

In order to probe the stability of the vaterite surface, geometry optimizations were performed in the presence of a continuum solvent model. This uses the COSMIC method as implemented in GULP[16,17], which is a variant of the COSMO methodology,[18] but with the constraint that the total induced charge on the solvent accessible surface (SAS) remains at a fixed integer value such that the electrostatics can be correctly handled within periodic boundary conditions. In the implicit solvent calculations, the dielectric constant was set to 78.4 for water, the solvent radius was 1.2 Å and a smoothing range of 0.2 Å was used in the construction of the SAS. The radii for Ca, C and O were set to 1.8885, 1.319 and 1.319 Å, respectively, based on fitting the hydration free energies of Ca^{2+} and CO_3^{2-}.

The interatomic interactions were described via Lennard-Jones (LJ and LJ-AB) and Buckingham potentials, as shown in Supplementary Equations 1-3, respectively.

$$E_{LJ} = 4\epsilon\left[\left(\frac{\sigma}{r}\right)^{12} - \left(\frac{\sigma^6}{r}\right)\right] \quad (1)$$

$$E_{LJ-AB} = \left(\frac{A}{r}\right)^{12} - \left(\frac{B}{r}\right)^{6} \quad (2)$$

$$E_{Buckingham} = A \cdot exp\left(\frac{-r}{\rho}\right) - \frac{C}{r^6} \quad (3)$$

The parameters derived for this work are reported in Supplementary Tables 3-5, with labelling of the atoms as show in Supplementary Figure 11.

Supplementary Discussions

Physical mechanism of the homochiral assembly of molecular enantiomers inducing the homochiral nanostructured chiral platelets in vaterite helicoids. Given that the vaterite nanostructured chiral platelets of homochiral vaterite helicoids are composed of subunit hexagonal nanoparticles[19], it is reasonable to consider that the modulation of homochiral assembly of Asp enantiomers on platelet chirality is under the control of the configuration of the subunit nanoparticles. From the thermodynamic point of view, the interference of a homochiral assembly domain of amino acid enantiomer incorporated into nanostructured materials can induce a strain attributable to a mismatch between the nanocrystalline structure and the homochiral assembly domain, but with opposite directions for L- and D-enantiomers[19-22]. For stabilization, an imperfect oriented attachment strategy (nano-tilting misalignment) between adjacent nanoparticles of chiral platelets is used to release this strain penalty, which induces homochiral counterclockwise and clockwise curved vaterite platelets in helicoids for L-and D-Asp, respectively[19]. For the disorganized heterochiral mixture, the opposite strains induced by L- and D-Asp can offset each other, which explains why oriented homochiral platelets were not formed on the vaterite structure in a racemic system. In the nonracemic

system, the homochiral domain-induced nanoparticle strain increases with increasing homochiral domain assembly size. When the homochiral assembly reaches the critical size (e.e.$_L$>5%) where the strain is larger than the force resulting from lattice mismatching between two vaterite adjacent nanoparticles, the nanoparticle tilting misalignment effect will be triggered to release the strain energy in the nonracemic system, and then homochiral platelets will form on the vaterite helicoid. With further increases in the homochiral assembly domain (*i.e.*, e.e.$_L$ increase), the dominance of this released energy is strengthened, which leads to the density increase in oriented chiral platelets of the homochiral helicoids, and which also explains why the density of the oriented platelets of the helicoids increases with the excess concentration of L-Asp (the concentration of L-Asp after neutralization by D-Asp) rather than with its absolute concentration in the nonracemic system (Fig. 3, and Supplementary Figure 2).

The behavior of mixed Asp enantiomers in an aqueous solution system. To exclude a possible influence of homotypic enantiomer interactions unrelated to the mineral surface that might occur initially in the aqueous mineral growth solution having added Asp, we examined the status of mixed amino acid enantiomers in aqueous solution system. Unlike dry solid samples of helicoid-incorporated Asp or pure Asp enantiomer crystal, the peaks of water completely covered the typical C-H peaks of amino acid in micro-Raman spectra (Supplementary Figure 7). From here, we then used circular dichroism (CD) and nuclear magnetic resonance (NMR) spectroscopy to examine the solution system. By CD, the relationship of the chiral ellipticity versus the concentration of pure enantiomer Asp or enantiomeric Asp excess were linear, and no chemical shift or broadening of any resonance appeared with increasing concentration of L-Asp in NMR spectroscopy (Supplementary Figure

6), which confirmed that no chiral amplification and no homochiral assembly of majority Asp enantiomers occurs in solution. Together, these data confirm that the assembly interactions between adjacent homochiral enantiomers occurs on the solid vaterite crystalline surface only rather than initially in aqueous solution, consistent with the notion that the mineral surface itself (lattice atoms) induces the assembly of homochiral Asp domains on vaterite[23,24].

Computational simulation

Vaterite surface and aqueous interface. Four charge-neutral surfaces with spacing *d*=3.6 Å (as identified in Ref 19) have been found within the hexagonal and the monoclinic basins of vaterite structures[25]. They are all micro-faceted, and three of these exhibit a tendency to dissolve in water after a few tens of ns of unbiased molecular dynamics simulation. One, (020) derived from the monoclinic basin, remains stable in water for over 2 μs; while we cannot exclude that aspartate or other experimental conditions could stabilize the surfaces that appear unstable, we have chosen to use the (020) in our simulations. Structurally, it is similar to the (110) surface derived from the hexagonal model that was used in a previous work[19]. Geometry optimization in implicit water confirms that this surface is stable and has a surface energy of 0.408 J/m^2. This compares to a value of 0.276 J/m^2 computed for the stable calcite basal plane using the same approach. The water residence time on the 3 symmetry-independent calcium surface sites is < 2 ns; since all our simulations are at least one order of magnitude longer, it is not necessary to bias-accelerate the water coordination on the surface when considering the binding of aspartate.

Adsorption of aspartate at the vaterite surface. At the experimental pH, the dominant

aspartic acid species in solution is Asp^{2-}, *i.e.* the fully deprotonated form. With the surface being very rough due to the micro-facets and with Asp^{2-} being a complex anion, having three functional groups with the potential to coordinate to Ca^{2+} as well as conformational flexibility, computing the binding energy is particularly challenging as numerous variables need to be considered (C-C-C-C torsional angle, distance of each functional group to the surface, D and L enantiomers, different surface sites). However, preliminary metadynamics simulation biased with respect to the C-C-C-C torsional angle of aspartate and the position of its center of mass with respect to the surface identifies the most likely binding geometry. We have then taken this arrangement and run unbiased molecular dynamics for up to 70 ns. L-Asp^{2-} stays bound to the surface all the time, whereas D-Asp^{2-} tends to leave the surface after 20 ns. This is not enough to claim that the surface is enantioselective, as different binding sites and configurations are possible and have not been fully explored in this work. Stronger binding is exhibited by both L- and D-Asp^{1-} (with a protonated amino group), which remain bound throughout the full length of the simulations. Given that our model is nonreactive, we are unable to investigate whether any proton exchange occurs at the binding sites. These simulations, however, provide at least qualitative evidence that aspartate anions bind to vaterite via at least a single well-defined minimum, though we cannot exclude the possibility of further bound configurations. The binding is also found to occur mostly through the carboxylate functional groups, as opposed to via the NH_2/NH_3^+ groups, as appropriate to the protonation state of the aspartate (Supplementary Figure 9).

To date, it has not been possible to compute the adsorption free energy of aspartate on the vaterite surface using metadynamics because of the large number of collective variables

required to unambiguously map the free energy landscape for this complex environment. Thus, to gain at least some initial insight we have used the configuration obtained from the metadynamics to perform a geometry optimization of aspartate when adsorbed in the presence of implicit solvent, as described previously for the clean surface. Based on this approach, the hydration energy for Asp^{2-} is -973 kJ/mol, which is reasonable agreement with the value from the force field model obtained by free energy perturbation (-1031 kJ/mol). The energy for the binding of L-Asp^{2-} to the vaterite surface is estimated to be -55.6 kJ/mol. While the absolute quantitative value is almost certainly overestimated due to the lack of explicit water and absence of a full treatment of entropic effects, this value at least suggests binding of aspartate to the surface. To place this value in context for the model, the energy of Asp^{2-} binding to a Ca^{2+} ion in implicit solvent (ion pair formation) is -25.4 kJ/mol if the same charge neutralizing constraint is applied as at the surface (or -35.1 kJ/mol if using the original COSMO formulation for nonperiodic species with an unconstrained SAS charge). As will be shown in the next section, this ion pairing binding energy represents a significant overestimate of the explicit force field value (since solvent-mediated binding is important for the ion pair). However, the important issue here is the qualitative conclusion that surface adsorption is favorable relative to ion pair formation in solution.

Cluster formation in explicit water. To support the hypothesis of Asp^{2-} cluster formation occurring at the interface with vaterite, and thus with the surface favoring their formation and homochiral organization, we have explored the solution speciation. Here we have performed metadynamics simulations where we progressively add ions to form a small cluster consisting of up to 2 formula units in explicit water. This results in two ion pairs (CaL-Asp, Ca D-Asp),

two 3-ion clusters ($CaL\text{-}Asp_2^{2-}$; $CaL\text{-}AspD\text{-}Asp^{2-}$), and two 4-ion clusters ($Ca_2L\text{-}Asp_2$; $Ca_2L\text{-}AspD\text{-}Asp$).

The pairing free energy profile of L-Asp^{2-} with Ca^{2+} is reported in Supplementary Figure 8a and shows weak binding (pairing free energy is -10.1 kJ/mol for the Ca^{2+}-α-carboxylate interaction, -9.7 kJ/mol for the Ca^{2+}-chain-carboxylate interaction and -11.0 kJ/mol when the distance between Ca^{2+} and the center of mass of L-Asp^{2-} is considered as the only collective variable), with both the carboxylate groups being involved in the formation of the ion pair. A strong preference exists for a solvent-shared rather than a direct contact configuration for the Ca^{2+}-chain-carboxylate interaction. The pairing free energy profile of D-Asp^{2-} with Ca^{2+} in water appears nearly identical to that of L-Asp^{2-} with Ca^{2+} and does not need additional discussion; the pairing free energy between D-Asp^{2-} and Ca^{2+} is -10.8 kJ/mol and the deviation from the value calculated for L-Asp^{2-} Ca^{2+} is well within thermal energy and therefore within the statistical uncertainty of the method. The formation of larger clusters appears less favorable (Supplementary Figure 8b,c), as the binding free energy of L- and D-Asp^{2-} to an ion pair is -2.8 and -3.2 kJ/mol, respectively. The binding free energy of a further Ca^{2+} ion to form a neutral 2 formula unit cluster is -11.4 kJ/mol for both the homochiral and the heterochiral clusters. Here, the distance between two calcium cations has been used as collective variable. From these numbers, we can derive that the association of two ion pairs as suggested in Supplementary Table 2 to form a homochiral and a heterochiral neutral cluster has a free energy of -3.2 and -3.8 kJ/mol. Again, the small difference between these numbers is largely within the uncertainty of the method, and so it is not possible to draw any conclusion as to whether there is a meaningful chiral effect. Despite *ab initio* calculations in vacuum showing a strong

preference for the formation of homochiral clusters (-28 kJ/mol for Asp^{2-}), this preference disappears in water. While a detailed analysis of the clusters' structures is beyond the aim of this study, and would require sampling along numerous collective variables, from comparison of the profile and the position of the minima in the plots with the ion pair case, we deduce that part of the Ca^{2+}-carboxylate interactions are mediated by water in a solvent-shared mode, so that these weakly-bound clusters are also highly hydrated.

These results demonstrate that calcium aspartate clusters, beyond ion pairs, are unlikely to form in solution at the experimental pH/concentration and that their weak association is nearly the same for homochiral and heterochiral clusters. From this, we deduce that in the experiments it is the presence of vaterite that determines the preference for the formation of homochiral clusters, as well as favoring the formation of any clusters.

Supplementary Figures below

Supplementary Figures

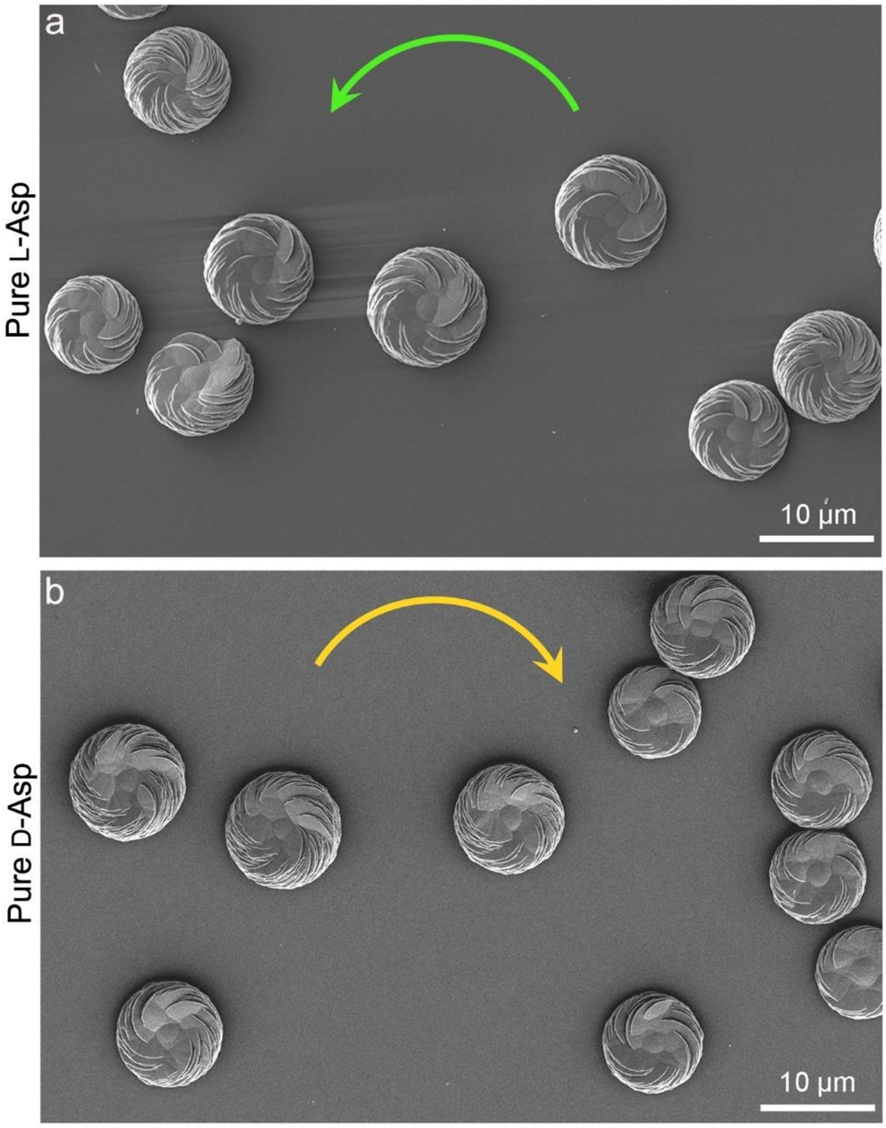

Supplementary Figure 1 | Homochiral vaterite helicoids induced by pure chiral Asp enantiomers. SEM images of homochiral vaterite helicoids grown in 20 mM L-Asp which have a counterclockwise spiraling morphology (green arrow, **a**), whereas in 20 mM D-Asp they have a clockwise spiraling morphology (yellow arrow, **b**).

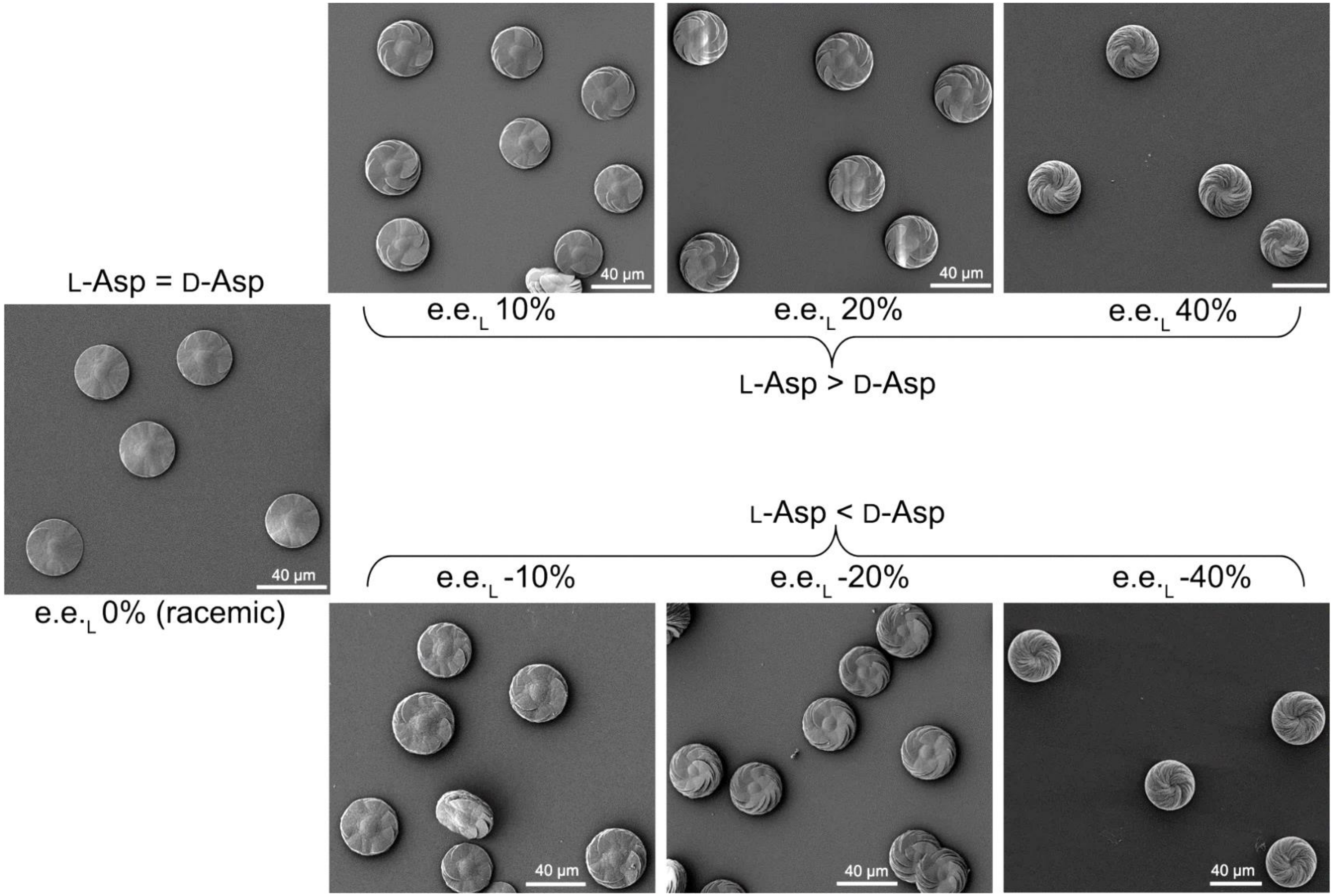

Supplementary Figure 2 | Uniformity of platelet orientation and density in chiral vaterite helicoids formed in Asp solutions. The density of the oriented platelets increases nonlinearly with increasing enantiomeric excess (e.e.) as shown in Supplementary Figure 5 in the Supplementary Materials. Under the racemic condition where the concentration of L-Asp is equal to that of D-Asp, chiral platelets almost never formed (left-most panel).

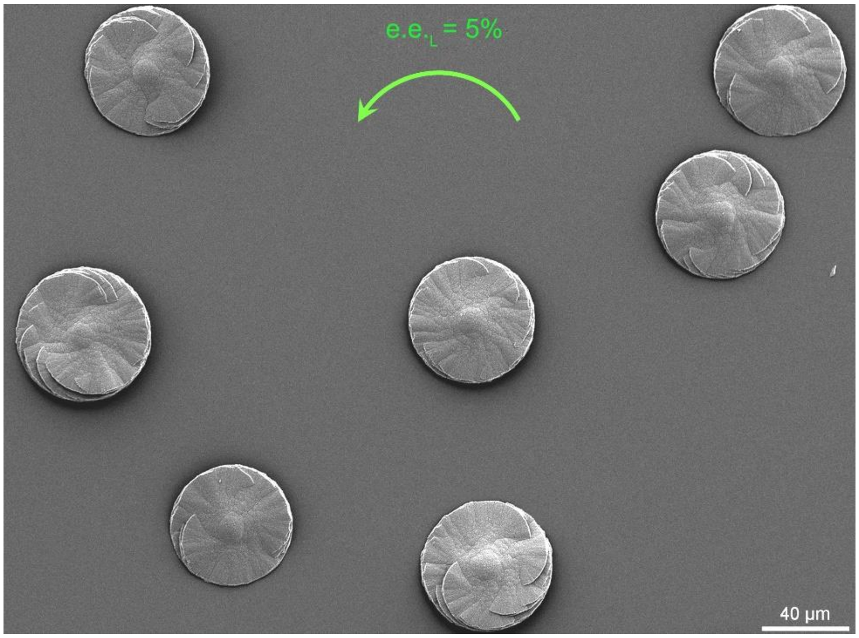

Supplementary Figure 3 | Homochiral vaterite helicoids induced in the presence of an imbalanced (nonracemic) mixture of Asp enantiomers with low enantiomeric excess. SEM images of chiral vaterite helicoids after growth in the presence of 20 mM total Asp enantiomer mixture consisting of 10.5 mM L-Asp and 9.5 mM D-Asp (e.e.$_L$ 5%), showing identical counterclockwise orientation of vaterite platelets (green arrow), but with low platelet density.

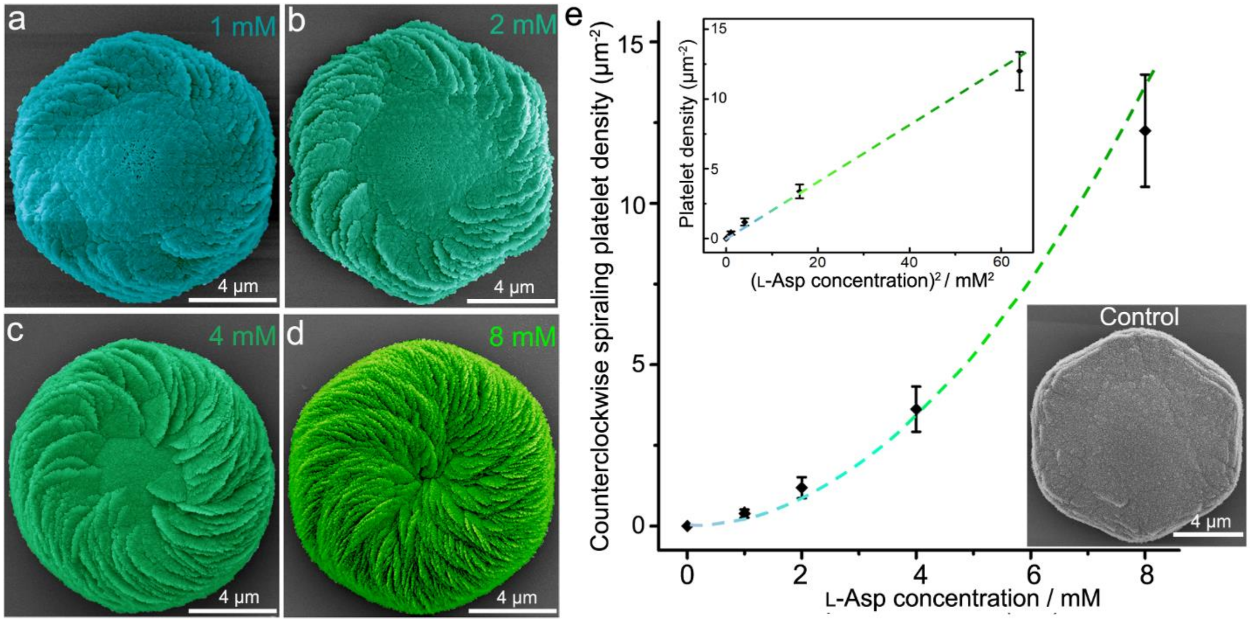

Supplementary Figure 4 | Relationship between the density of counterclockwise platelets in chiral vaterite helicoids and the concentration of pure L-Asp. (**a-d**) Representative SEM images of chiral vaterite helicoids showing an increase in counterclockwise-oriented platelets with increasing concentration of enantiomer (pseudocolored green) in a pure L-Asp system. (**e**) Plot showing a nonlinear increase in the density of counterclockwise-oriented platelets with increasing concentration of pure L-Asp. The inset SEM image shows typical achiral hexagonal vaterite structure in the absence of additives showing no chirality, and the inset plot illustrates the linear proportionality relationship between the density of homochiral platelets density (D) of the helicoids and the square of the concentration of L-Asp (θ_{L}):

$$D = a_{\mathrm{pure}}(\theta_{\mathrm{L}})^2$$

where a_{pure} is constant of 0.2 μm^-2 mM^-2. Error bar represents standard error of mean.

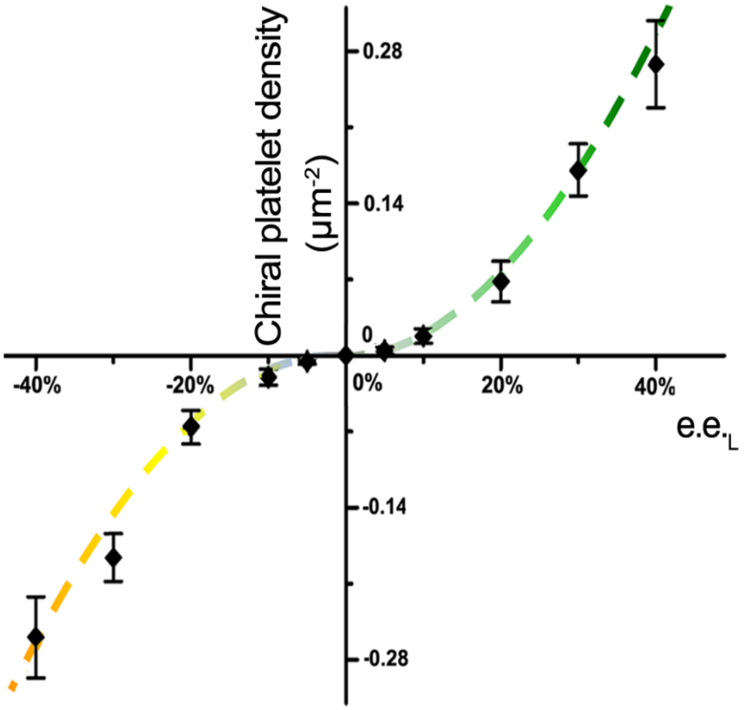

Supplementary Figure 5 | Relationship between the density of counterclockwise platelets in chiral vaterite helicoids and the enantiomeric excess (e.e.) in a mixed system. Plot showing a nonlinear increase in homochiral oriented platelets with increasing e.e.L, having a linear proportionality relationship between the density of homochiral platelets and the square of enantiomeric excess $(e.e._L)^2$ as shown in Fig. 3g in main text ("+" is arbitrarily assigned to counterclockwise-oriented platelets induced by L-Asp, and "-" to clockwise-oriented platelets induced by D-Asp). Error bar represents standard error of mean.

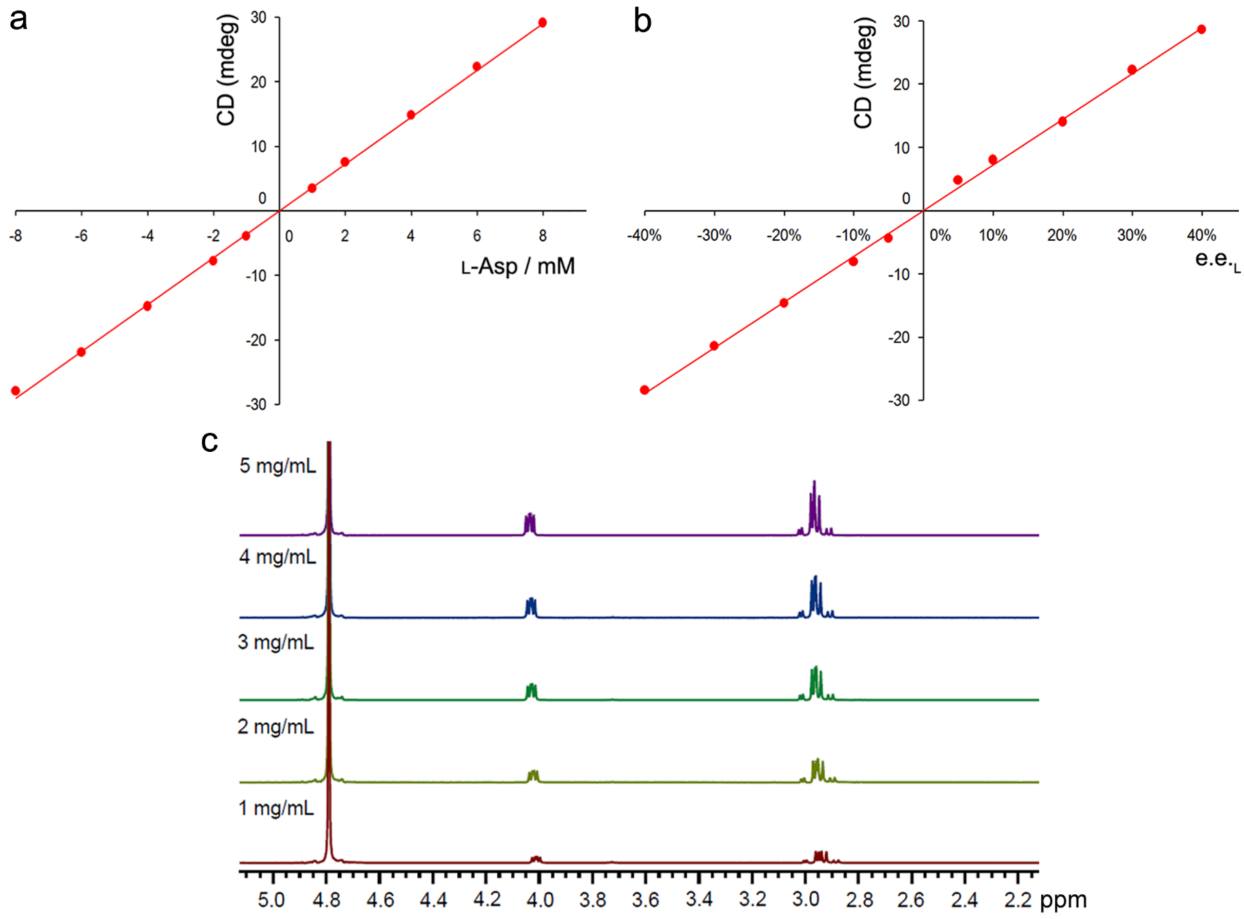

Supplementary Figure 6 | No assembly of Asp enantiomer in solution without vaterite. (**a**) Ellipticity of pure L- or D-Asp in water measured by circular dichroism (CD) at 200 ± 2 nM at different molar concentrations ("+" is arbitrarily assigned to L-Asp, and "-" to D-Asp). (**b**) Ellipticity of the mixture of L-Asp and D-Asp with different enantiomeric excess of L-Asp ($e.e._{L}$). In both plots (a) and (b), ellipticity of the solution increases linearly with the molar concentration of L-Asp, *i.e.*, the increase of pure enantiomer concentration or enantiomeric excess, demonstrating that no self-assembly occurred in water without vaterite. (**c**) NMR spectra of L-Asp in D_2O, which shows no chemical shift and no broadening of any resonance with increasing concentration of L-Asp, indicating no self-assembly of L-Asp in water.

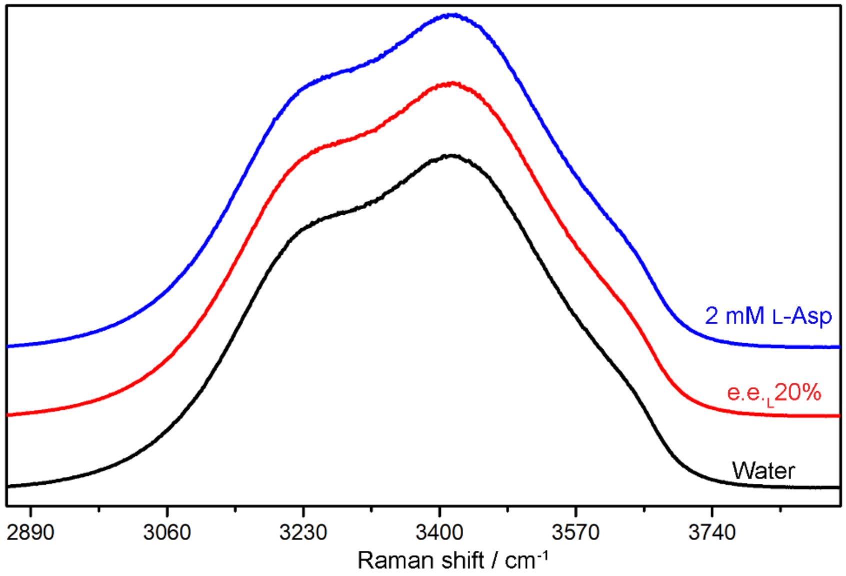

Supplementary Figure 7 | Overlapping of C-H stretching bands of Asp enantiomer with O-H stretching bands of water in aqueous solution. Micro-Raman spectra of 2 mM L-Asp in water, a mixture of L- and D-Asp enantiomers (total Asp concentration equals 20 mM) at e.e.$_{L}$ 20%, and pure water. All micro-Raman spectra are the same, and the peaks of O-H stretching bands of water completely covered the typical peaks of C-H stretching bands of Asp.

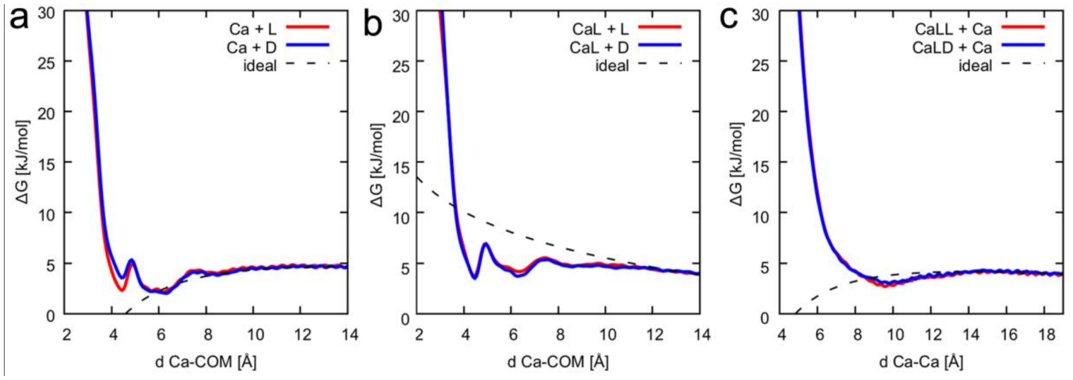

Supplementary Figure 8 | Free energy profiles for (**a**) Ca^{2+} and L-and D-Asp^{2-} ion pair formation; (**b**) L- and D-Asp^{2-} binding to the CaL-Asp ion pair (CaL); and (**c**) Ca^{2+} binding to CaL-Asp_2^{2-} (CaLL) and CaL-AspD-Asp^{2-} (CaLD) in explicit water. (a) and (b) are projected along the distance between calcium and the center of mass of aspartate; (c) along the distance between the two calcium cations. The theoretical free energy profile for two point particles with charge +2 and -2 (a and c) and 0 and -2 (b) as a function of their distance in water is shown on all plots as a dashed line. Convergence to this asymptote in the long-range limit is used to validate the simulations.

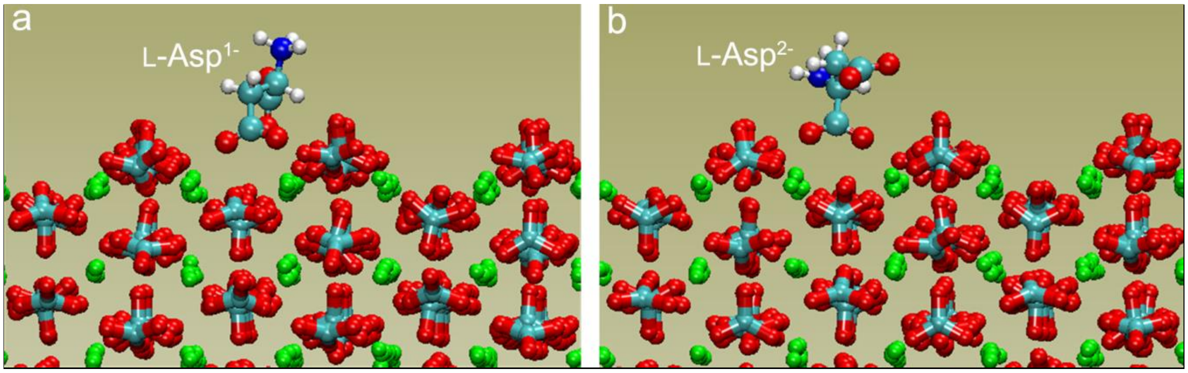

Supplementary Figure 9 | Binding of L-Asp^{1-} (**a**) and L-Asp^{2-} (**b**) to the (020) surface of monoclinic vaterite after 70 ns of unbiased molecular dynamics. Calcium atoms are colored in green, carbon atoms in cyan, oxygen and hydrogen atoms in red and white, respectively, and nitrogen in blue. Water is omitted for the sake of clarity.

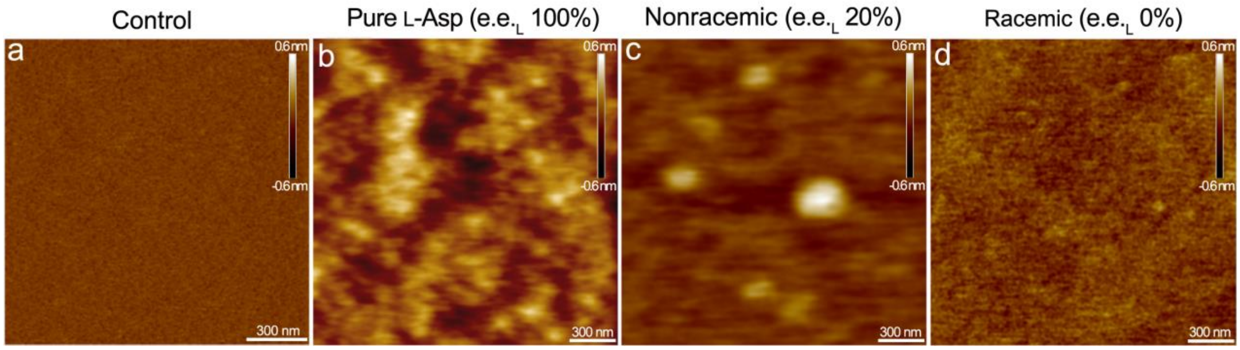

Supplementary Figure 10 | Atomic force microscopy (AFM) of the aggregation of homochiral L-Asp on cleaved calcite in the presence of minority D-Asp. Tapping mode AFM images of freshly cleaved calcite crystal surfaces in the absence of amino acids (**a**), and in the presence of pure L-Asp (e.e.$_L$ 100%) (**b**), nonracemic Asp enantiomer mixture (e.e.$_L$ 20%) (**c**) and racemic Asp enantiomer mixture (e.e.$_L$ 0%) (**d**), respectively. Clearly, large and abundant homochiral domains (bright areas in panel a) of L-Asp form on the calcite crystal surface in the absence of minority D-Asp (b). However, the presence of the minority D-Asp interferes with the formation of homochiral domains of majority L-Asp (c), and almost no large aggregation domains form in the racemic system having equal L-Asp and D-Asp.

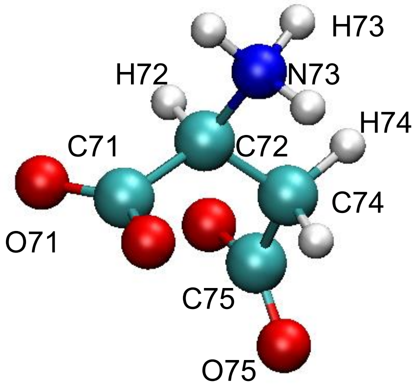

Supplementary Figure 11 | Labelling of atoms for the Asp^{1-} anion. Asp^{2-} is the same, but with only two H73. H atoms are colored in white, nitrogen is blue, carbon atoms are cyan and oxygen atoms red. The atoms of water have been labelled as O2 and H2; those in carbonate as C4 and O4.

Supplementary Tables

Supplementary Table 1: L- and D-Asp concentration from acid-dissolved chiral vaterite helicoids synthesized in a nonracemic mixed system

	L-Asp concentration (mM)	D-Asp concentration (mM)	Ratio of incorporated L- and D-Asp
Counterclockwise helicoids synthesized in solution with 6:4 ratio of L- and D-Asp (triplicate samples)	3.22	2.14	6.02:4.00
	3.34	2.24	5.96:4.00
	3.14	2.08	6.04:4.00
Clockwise helicoids synthesized in solution with 4:6 ratio of L- and D-Asp (triplicate samples)	2.10	3.17	4.00:6.04
	2.18	3.26	4.00:5.98
	2.22	3.33	4.00:6.00

Supplementary Table 2: Association free energies (ΔG, kJ/mol) of ions to form homochiral and heterochiral clusters in aqueous solution. L and D stand for L- and D-Asp^{2-} anions, respectively

						X=L	X=D
CaL		→	Ca^{2+}	+ L^{2-}	$-\Delta G_1$	11.0	11.0
CaX	+ L^{2-}	→	$CaXL^{2-}$		ΔG_2	-2.8	-3.2*
$CaXL^{2-}$	+ Ca^{2+}	→	Ca_2XL		ΔG_3	-11.4	-11.4
CaL	+ CaY	→	Ca_2XL		$-\Delta G_1 + \Delta G_2 + \Delta G_3$	-3.2	-3.8

* We assume that D binds to CaL with the same binding energy as L to CaD.

Supplementary Table 3: E_{LJ} (Supplementary Equation 1) coefficients for the intra-molecular interactions of Asp^{1-} and Asp^{2-}, their interaction with water and with carbonate. Labels as in Supplementary Figure 11; a and b indicate Asp^{1-} and Asp^{2-}, respectively, when the parameters for the two anions are different.

		ε [eV]	σ [Å]
Asp Intra-molecular			
C71	C71	0.00288	3.58118
C71	C72	0.00288	3.58118
C71	C74	0.00288	3.58118
C71	C75	0.00288	3.58118
C71	H72	0.00188	2.91541
C71	H74	0.00188	2.91541
C71	N73	0.00437	3.35146
C71	O71	0.00717	3.06654
C71	O75	0.00717	3.06654
C72	C72	0.00288	3.58118
C72	C74	0.00288	3.58118
C72	C75	0.00288	3.58118
C72	H72	0.00188	2.91541
C72	H74	0.00188	2.91541
C72	N73	0.00437	3.35146
O71	C72	0.00717	3.06654
C72	O75	0.00717	3.06654
C74	C74	0.00288	3.58118
C74	C75	0.00288	3.58118
H72	C74	0.00188	2.91541
C74	H74	0.00188	2.91541
N73	C74	0.00437	3.35146
O71	C74	0.00717	3.06654
C74	O75	0.00717	3.06654
C75	C75	0.00288	3.58118
H72	C75	0.00188	2.91541
H74	C75	0.00188	2.91541
N73	C75	0.00437	3.35146
O71	C75	0.00717	3.06654
C75	O75	0.00717	3.06654
H72	H72	0.00123	2.37341
H72	H74	0.00123	2.37341
H72	N73	0.00285	2.72839
O71	H72	0.00468	2.49644
H72	O75	0.00468	2.49644
H74	H74	0.00123	2.37341
N73	H74	0.00285	2.72839
O71	H74	0.00468	2.49644
H74	O75	0.00468	2.49644
N73	N73	0.00663	3.13647
O71	N73	0.00152	3.98580
N73	O75	0.00152	3.98580
O71	O71	0.01788	2.62585
O71	O75	0.01788	2.62585
O75	O75	0.01788	2.62585
water-Asp			

O2	C71	0.00440	3.36701
O2	C72	0.00440	3.36701
O2	C74	0.00440	3.36701
O2	C75	0.00440	3.36701
O2	H72	0.00288	2.74105
O2	H74	0.00288	2.74105
O2	N73a	0.00332	3.47115
O2	O71a O75a	0.00513	3.27240
O2	N73b O71b	0.00513	3.27240
O2	O75b H73b	0.00332	3.37115
O2		0.00513	3.17240
O2		0.00513	3.17240
H2		0.00123	2.45000
CO_3^{2-} - Asp			
C4	C72	0.00288	3.58118
C4	H72	0.00188	2.91541
C4	C74	0.00288	3.58118
C4	H74	0.00188	2.91541
C4	N73	0.00437	3.35146
O4	C72	0.00717	3.06654
O4	H72	0.00468	2.49644
O4	C74	0.00717	3.06654
O4	H74	0.00468	2.49644
O4	N73	0.00152	3.98580
O4	O71	0.01788	2.62585
O4	O75	0.01788	2.62585
C4	C71	0.00288	3.58118
C4	C72	0.00288	3.58118

Supplementary Table 4: $E_{LJ\text{-}AB}$ (Supplementary Equation 2) and $E_{Buckingham}$ (Supplementary Equation 2) and coefficients for the interactions of Asp^{1-} and Asp^{2-} with calcium in water. Labels as in Supplementary Figure 11.

LJ-AB		A [eV Å^{12}]	B [eV Å^6]	
Ca	C72	12000.00	0.0	
Ca	C74	12000.00	0.0	
Buckingham		A [eV]	ρ [Å]	C [eV Å^6]
Ca	O71	2388.4841	0.271511	0.0
	O75	2388.4841	0.271511	0.0
Ca	N73	22878.8	0.215023	0.0
Ca				

Supplementary Table 5: Partial charges on atoms. Labels as in Supplementary Figure 11; "a" refers to Asp^{1-} and "b" to Asp^{2}.

C71a,b	0.499
O71a,b	-0.702
C72a,b	0.198
H72a,b	0.024
N73a	-0.449
N73b	-0.923
H73a	0.326
H73b	0.226
C74a,b	-0.241
H74a,b	0.070
C75a,b	0.715
O75a,b	-0.730

Supplementary References

1. Plimpton, S. Fast parallel algorithms for short-range molecular dynamics. *J. Comput. Phys.,* **117**, 1–19 (1995).
2. Raiteri, P., Laio, A., Gervasio, F. L., Micheletti, C. & Parrinello, M. Efficient reconstruction of complex free energy landscapes by multiple walkers metadynamics. *J. Phys. Chem. B*, **110**, 3533–3539 (2006).
3. Barducci, A., Bussi, G. & Parrinello, M. Well-tempered metadynamics: A smoothly converging and tunable free-energy method. *Phys. Rev. Lett.* **100**, 20603 (2008).
4. Tribello, G. A., Bonomi, M., Branduardi, D., Camilloni, C. & Bussi, G. Computer physics communications. *Comput. Phys. Commun.* **185**, 604–613 (2014).
5. Hutter, J., Iannuzzi, M., Schiffmann, F. & VandeVondele, J. CP2K: Atomistic simulations of condensed matter systems," *Comput. Mol. Sci.* **4**, 15−25 (2014).
6. VandeVondele, J., Krack, M., Mohamed, F., Parrinello, M., Chassaing, T. & Hutter, J. Quickstep: fast and accurate density functional calculations using a mixed gaussian and plane waves approach, *Comput. Phys. Commun.*, 167, 103−128 (2005).
7. Goedecker, S., Teter, M. & Hutter, J. Separable dual-space gaussian pseudopotentials," *Phys. Rev. B*, **54**,

1703−1710, 1996.

8. VandeVondele, J. & Hutter, J. An efficient orbital trans-formation method for electronic structure calculations," *J. Chem. Phys.*, 118, 4365−4369, 2003.
9. Wang, J., Român-Peŕez, G., Soler, J. M., Artacho, E. & Fernańdez-Serra, M. V. Density, structure, and dynamics of water: the effect of van der Waals interactions," *J. Chem. Phys.*, **134**, 024516 (2011).
10. DiStasio Jr., R. A., Santra, B., Li, Z., Wu, X. & Car, R. The individual and collective effects of exact exchange and dispersion interactions on the Ab initio structure of liquid water", *J. Chem. Phys*, **141**, 084502 (2014).
11. Neese, F. The ORCA program system. *Comput. Mol. Sci.*, 2, 73−78(2012).
12. Oostenbrink, C., Villa, A., Mark, A. E. & van Gunsteren, W. F. A biomolecular force field based on the free enthalpy of hydration and solvation: the GROMOS force-field parameter sets 53A5 and 53A6. *J. Comput. Chem.*, **25**, 1656–1676 (2004).
13. Malde, A. K., Zuo, L., Breeze, M., Stroet, M., Poger, D., Nair, P. C., Oostenbrink, C. & Mark, A .E. An automated force field topology builder (ATB) and repository: version 1.0. *J. Chem. Theory Comput.*, **7**, 4026–4037 (2011).
14. Wu, Y., Tepper, H. & Voth, G. A. Flexible simple point-charge water model with improved liquid-state properties. *J. Chem. Phys.*, **124**, 024503 (2006).
15. Raiteri, P., Demichelis, R. & Gale, J. D. Thermodynamically consistent force field for molecular dynamics simulations of alkaline-earth carbonates and their aqueous speciation. *J. Phys. Chem. C*, **119**, 24447–24458 (2015).
16. Gale J. D. & Rohl, A. L. An efficient technique for the prediction of solvent-dependent morphology: the COSMIC method. *Mol. Simul.*, **33**, 1237–1246 (2007).
17. Gale J. D. & Rohl, A. L. The general utility lattice program," *Mol. Simul.*, **29**, 291–341 (2003).
18. Klamt, A. & Schueuermann. G. COSMO: a new approach to dielectric screening in solvents with explicit expressions for the screening energy and its gradient. *J. Chem. Soc. Perkin Trans.* **2**, 799–805 (1993).
19. Jiang, W. *et al.* Chiral acidic amino acids induce chiral hierarchical structure in calcium carbonate. *Nat. Commun.* **8,** 15066 (2017).
20. Jiang, W., Pacella, M. S., Vali, H., Gray, J. J. & McKee, M. D. Chiral switching in biomineral suprastructures induced by homochiral L-amino acid. *Sci. Adv.* **4,** eaas9819 (2018).
21. Jana, S., de Frutos, M., Davidson, P. & Abécassis, B. Ligand-induced twisting of nanoplatelets and their self-assembly into chiral ribbons. *Sci. Adv.* **3,** e1701483 (2017).
22. Bertolotti, F. *et al.* Crystal symmetry breaking and vacancies in colloidal lead chalcogenide quantum dots. *Nat. Mater.* **15,** 987–994 (2016).
23. Hazen, R. M. & Sholl, D. S. Chiral selection on inorganic crystalline surfaces. *Nature Mater.* **2,** 367–374 (2003).

24. Lorenzo, M. O. & Baddeley, C. J., Muryn, C. & Raval, R. Extended surface chirality from supramolecular *assemblies of adsorbed chiral mo*lecules. *Nature* **404,** 376–379 (2000).
25. Demichelis, R., Raiteri, P., Gale, J. D., Dovesi, R. The multiple structure of vaterite. *Cryst. Growth Des.,* **13**, 2247–2251 (2013).
